# Hair tourniquet syndrome

**DOI:** 10.4103/0256-4947.67088

**Published:** 2010

**Authors:** Yunsur Cevik, Cemil Kavalci

**Affiliations:** a From the Ministry of Health, Department of Emergency, Ankara Atatürk Training and Research Hospital, Ankara, Turkey; b From the School of Medicine-Department of Emergency, Trakya University, Edirne, Turkey

## Abstract

The hair tourniquet syndrome is a rare disorder. This syndrome has been described as involving the fingers, the toes and even the genitals. We report a case of hair tourniquet syndrome affecting multiple toes of an infant. After the hair fiber was removed there was a fast healing period and no signs of tissue necrosis were seen. The prompt diagnosis and treatment of the condition is vital to attain a good outcome and prevent further harm to the child.

A 4-month-old girl presented for review of persistent erythema and swelling affecting the left third and fifth toes. There was no history of similar problems. On inspection, the third and fifth toes of his left foot were red, swollen and tender with constriction rings at the level of middle phalanges (Figure 1). On examination, using loupe magnification, several tightly entwined hairs were found in the constrictions of the left third and fifth toes. These were cut and removed with fine forceps. The color and swelling improved overnight and the child was discharged home in the morning. No further problems were reported 2 weeks following this episode.

**Figure 1:**
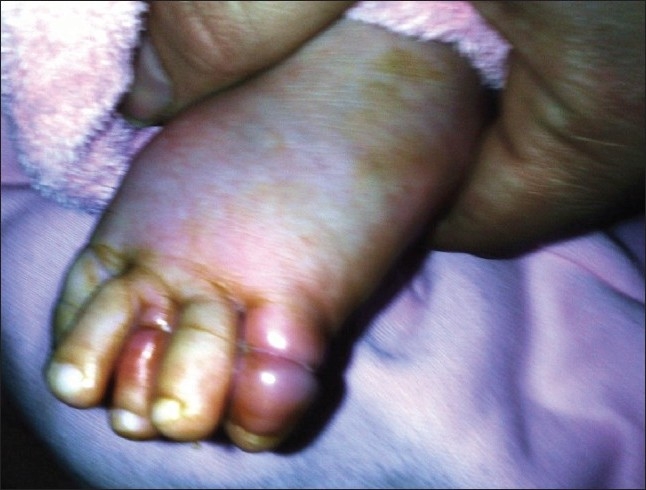
Photo of left foot showing erythema and edema distal to hair-toe tourniquets at the middle phalanges of the third and fifth toe.

## DISCUSSION

The hair tourniquet (HT) syndrome is a rare disorder. This syndrome has been described to involve the fingers, the toes and even the genitals.^1^,^2^ The majority of cases of HT syndrome have been reported in infants of less than 2 months of age.^3^ Although most cases are felt to be accidental, child abuse must be considered in selected cases. HT syndrome involving the toes occurs during the time period when postpartum mothers are experiencing increased hair loss. This condition is also known as toe tourniquet syndrome.^4^ Circumferential digital strangulation impairs lymphatic and venous drainage causing distal edema. Further obstruction may cause arterial occlusion and ischemic injury.^5^ Prolonged ischemic injury leads to tissue necrosis and ultimately autoamputation. Treatment is prompt removal of the constricting hair or fiber. It can usually be removed by direct inspection. Surgical exploration is mandatory if doubt persists as to the completeness of removal, especially as the hair cuts through the skin and becomes invisable.^1^,^5^

Toe tourniquet syndrome is a rare and dangerous but a preventable condition of young infants. The prompt diagnosis and treatment of the condition is vital to attain a good outcome and prevent further harm to the child. New parents should be warned that if excessive hair loss should occur, then their infant should be carefully checked on a regular basis to make sure that no hairs are becoming entangled in the fingers or toes.
